# MUC1 *O*-glycosylation contributes to anoikis resistance in epithelial cancer cells

**DOI:** 10.1038/cddiscovery.2017.44

**Published:** 2017-07-17

**Authors:** Tushar Piyush, Jonathan M Rhodes, Lu-Gang Yu

**Affiliations:** 1 Gastroenterology Unit, Department of Cellular and Molecular Physiology, Institute of Translational Medicine, University of Liverpool, Liverpool L69 3GE, UK

## Abstract

Anoikis is a fundamental cellular process for maintaining tissue homeostasis. Resistance to anoikis is a hallmark of oncogenic epithelial–mesenchymal transition and is a pre-requisite for metastasis. Previous studies have revealed that the heavily glycosylated mucin protein MUC1, which is overexpressed in all types of epithelial cancer cells, prevents anoikis initiation in response to loss of adhesion. This effect of MUC1 is largely attributed to its extracellular domain that provides cell surface anoikis-initiating molecules with a ‘homing’ microenvironment. The present study investigated the influence of *O*-glycosylation on MUC1 extracellular domain on MUC1-mediated cell resistance to anoikis. It shows that stable suppression of the Core 1Gal-transferase (C1GT) by shRNA substantially reduces *O*-glycosylation in MUC1-positively transfected human colon cancer HCT116 cells and in high MUC1-expressing SW620 cells. Suppression of C1GT significantly increased anoikis of the MUC1-positive, but not MUC1-negative, cells in response to suspended culture. This effect was shown to be associated with increased ligand accessibility to cell surface anoikis-initiating molecules such as E-cadherin, integrin*β*1 and Fas. These results indicate that the extensive *O*-glycosylation on MUC1 extracellular domain contributes to MUC1-mediated cell resistance to anoikis by facilitating MUC1-mediated prohibition of activation of the cell surface anoikis-initiating molecules in response to loss of cell adhesion. This provides insight into the molecular mechanism of anoikis regulation and highlights the importance of cellular glycosylation in cancer progression and metastasis.

## Introduction

Anoikis is a special apoptotic process that occurs in cells in response to loss of adhesion to the extracellular matrix.^1,2^ It is a fundamental cellular process for maintaining tissue homeostasis by removing displaced epithelial/endothelial cells and thus preventing them from seeding to inappropriate sites.

Anoikis initiation starts from the cell surface through activation of the cell surface anoikis-initiating molecules such as integrins and death receptors. Loss of cellular adhesion to the extracellular matrix induces changes (for example, conformational changes, oligomerization or ligand ligation) of the cell surface anoikis-initiating molecules which initiate a series of events leading to activation of either the caspase-8-mediated extrinsic apoptosis signalling pathway or the mitochondrial-mediated intrinsic apoptosis signalling pathway and consequent triggering of apoptosis.^3–5^ For example, loss of the cell surface integrin engagement with the extracellular matrix induces integrin conformational changes and the formation of oligomers that trigger inactivation of the pro-survival cell signalling such as those mediated by FAK and PI3K, leading to activation of the mitochondrial-mediated apoptosis signalling. Loss of cell–matrix contact induces ligation of the cell surface death receptors with their ligands, resulting in release of pre-caspase-8 from death receptor/FADD complex and subsequent activation of caspase-8-mediated extrinsic apoptosis signalling pathway. Resistance to anoikis is a hallmark of oncogenic epithelial–mesenchymal transition^6^ and metastasis^7^ and is a pre-requisite for metastasis.^8,9^

MUC1 is a large (MW up to 500 kDa) and heavily glycosylated transmembrane protein that is expressed exclusively on the apical side of normal epithelial cells and protrudes up to 10-times further than other typical cell surface molecules above the cell surface.^10^ It consists of a large and heavily glycosylated (up to 50% of the MUC1 molecular weight) extracellular domain, a transmembrane region and a short cytoplasmic tail. MUC1 is overexpressed up to 10-fold in epithelial cancer cells,^11^ where it also becomes expressed over the entire cell surface.^12^ Overexpression of MUC1 is closely associated with high metastatic potential and poor prognosis in cancer patients^13,14^ and is a much-studied therapeutic target in cancer therapy.^15^

In previous studies we found that overexpression of MUC1 prevents initiation of anoikis of epithelial cancer cells in response to loss of cell adhesion.^16^ We showed that this effect of MUC1 is attributed to both the MUC1 intracellular and extracellular domains but a predominate influence comes from the MUC1 extracellular domain. This prevents activation of the cell surface anoikis-initiating molecules in response to loss of cell adhesion, possibly by providing them a ‘homing’ microenvironment due to its large and elongated structure that protrudes over the cell surface. As the MUC1 extracellular domain is heavily modified by *O*-linked mucin type glycans, we have speculated that these *O*-linked sugar chains may play a role in MUC1-mediated cell resistance to anoikis upon loss of cell adhesion.

This study investigated the role of *O*-glycosylation on the MUC1 extracellular domain in MUC1-mediated resistance to anoikis. We used a shRNA strategy to shorten the MUC1 *O*-glycans by suppressing the Core 1Gal-transferase (C1GT, T-synthase) activity. C1GT is a key glycosyltransferase in the biosynthesis of *O*-linked mucin type glycans.^17^ It is responsible for the formation of the Core 1 structure (Gal*β*1,3GalNAc*α*-, T or TF antigen),^18–20^ which is overexpressed by over 90% of cancer cells^21^ and is a natural ligand of the galactoside-binding galectins.^12,22^ The formation of TF acts as a structural base for further elongation of the sugar chains to form other Core-1-related complex (for example, Core-2-associated) glycans.^23^ Although Core 1 formation is not directly involved in the biosynthesis of other complex (for example, Core-4-related) glycans, Core-1-related glycans have been reported to be the predominate structures of MUC1 *O*-glycosylation in breast cancer.^24^ We show in this study that stable shRNA suppression C1GT reduces MUC1 *O*-glycosylation and substantially reduces MUC1-mediated cell resistance to anoikis in human colon cancer cells.

## Results

### Generation of C1GT stable suppression in MUC1-positive and -negative human colon cancer HCT116 cells

**Figure 1:**
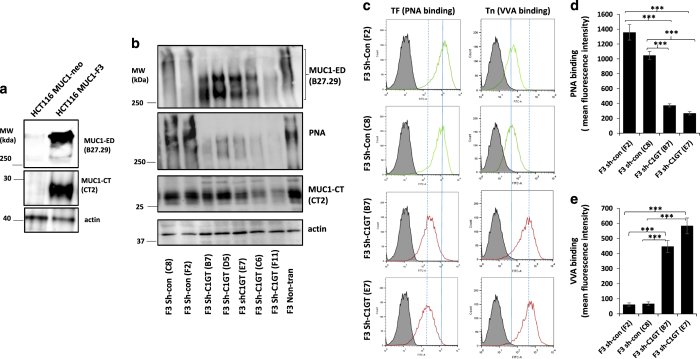
shRNA C1GT suppression in MUC1-expressing HCT116. MUC1 expression in HCT116 cells stably transfected with MUC1 (HCT116-F3) or control vector (HCT116-neo) were measured by immunoblotting with antibody B27.29 against the MUC1 extracellular domain (MUC1-ED), antibody CT2 against the MUC1 cytoplasmic tail/domain (MUC1-CT) or actin (**a**). MUC1-positive F3 cells were stably transfected with shRNA C1GT or control shRNA and a number of single-cell colonies were analysed by immunoblotting (**b**) with antibodies against MUC1 (B27.29 and CT2) or by biotin-PNA. The cells were also assessed for cell surface TF and Tn expressions by PNA and VVA binding by flow cytometry (**c**), solid and dotted lines show the positions of peak PNA binding without and with shRNA C1GT suppression, respectively. Dotted line shifted to the left or right of the solid line indicates reduction or increase of molecule expression, respectively). The mean florescence intensity of PNA and VVA cell surface binding were shown in **d** and **e**. Data in **d** and **e** are expressed as mean±S.D. florescent intensity from duplicate determinations of two experiments, ***P<0.001.

MUC1-positively (HCT116^MUC1-F3^, F3) and –negatively (HCT116^MUC1-neo^, Neo) transfected HCT116 cells were generated previously^16^ and their positive and negative MUC1 expression is also confirmed here (Figure 1a).

To determine the role of MUC1 *O*-glycosylation on MUC1-associated cell resistance to anoikis, we first generated C1GT stably suppressed cells using shRNA in both MUC1-positive and –negative cells. After transfection of the cells with shRNA C1GT or control shRNA, a number of single-cell colonies were obtained. Expressions of TF, Tn (*N*-acetyl-galactosamine*α*-, GalNAc-*α*) and MUC1 in these cells were analysed by lectin/immunoblotting using TF-binding peanut agglutinin (PNA), *N*-acetyl-galactosamine-binding Vicia Villosa lectin (VVA) and antibody against the MUC1 extracellular domain B27.29 and antibody against the MUC1 cytoplasmic domain CT-2. In comparison to the control shRNA transfected cells (C8, F2) and to the non-transfected cells, five single-cell colonies (B7, D5, E7, C6 and E11) from the shRNA C1GT transfected F3 cells showed various degrees of C1GT suppression as illustrated by the substantial (from above 400 to ~<300 kDa) reduction of the MUC1 extracellular domain molecular weight (as a result of reduced glycosylation) and reduction (45–73%) of TF expression (PNA binding; Figure 1b). Two of the more C1GT-suppressed colonies (E7 and B7, 66 and 73% reduction of PNA binding, respectively) were further analysed for cell surface TF and Tn expressions (PNA and VVA binding) by flow cytometry (Figures 1c–e). In comparison to control sh-con F2 cells, shRNA transfected sh-C1GT B7 and E7 cells showed 73 and 80%, respectively, reduction of cell surface TF structure. When compared to the control sh-con C8 cells, cell surface TF expression was reduced by 64 and 74%, respectively, in the shRNA transfected sh-C1GT B7 and E7 cells. A 623 and 845% increase of cell surface Tn structure was seen in sh-C1GT B7 and E7 cells, respectively, when compared to sh-con F2, and a 559 and 761% increase when compared to sh- con C8, respectively (Figures 1c and e). These results show that shRNA C1GT suppression has substantially reduced MUC1 *O*-glycans in these cells.

**Figure 2:**
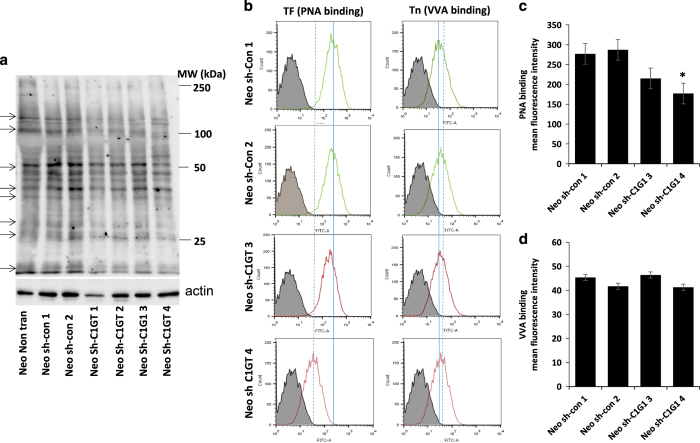
shRNA suppression of C1GT in MUC1-negative HCT116 cells. MUC1-negative (neo) cells were transfected with shRNA C1GT or control shRNA and a number of single-cell colonies were selected and assessed for TF expression by PNA blotting with biotin-PNA (**a**). A number of proteins show reduced TF expression (PNA binding) (arrowed). Cell surface TF and Tn expressions was assessed by biotin-PNA and biotin-VVA and analysed by flow cytometry (**b**, solid and dotted lines show the positions of peak PNA binding without and with shRNA C1GT suppression, respectively. Dotted line shifted to the left or right of the solid line reveals reduction or increase of molecule expression, respectively). The mean florescence intensity of PNA and VVA cell surface binding were shown in **c** and **d**. Data in **c** and **d** are expressed as mean±S.D. florescent intensity from duplicate determinations of two experiments, *P<0.05.

A similar shRNA C1GT approach was applied to the MUC1-negative Neo cells. A number of single-cell colonies (sh-C1GT1-4) from the cells treated with shRNA C1GT demonstrated reduction of TF structure (PNA binding) in comparison to the control shRNA-treated cells (sh-con1-4) when analysed by PNA blot (Figures 2a and b), suggesting successful shRNA C1GT suppression. Flow cytometry analysis also showed reduction of cell surface TF expression of the shRNA C1GT suppressed cells (Figures 2b and c), confirming C1GT suppression in these cells. Little change of Tn expression (VVA binding) was however observed in response to shRNA C1GT or control shRNA treatment in these cells (Figures 2b and d).

### Suppression of C1GT in MUC1-positive, but not –negative, cells increases anoikis

**Figure 3:**
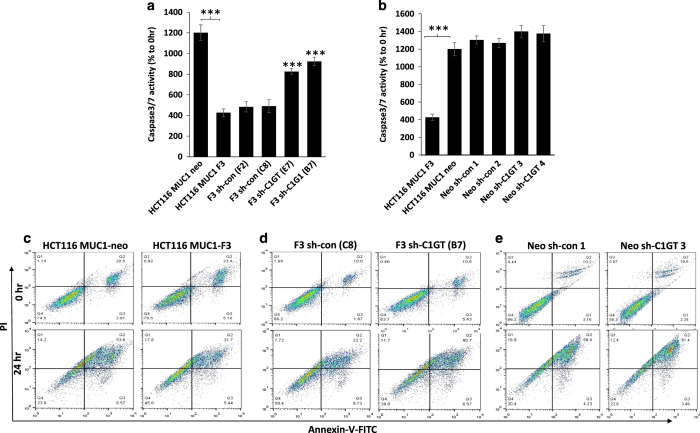
Effects of MUC1 expression and of shRNA C1GT suppression on anoikis in MUC1-positive and -negative cells. ShRNA control and shRNA C1GT transfected cells from MUC1-positive (F3) and negative (neo) cells were cultured in suspension. The cellular caspase 3/7 activities (**a** and **b**) or Annexin-V cell surface binding (**c**–**e**) in the transfected (**a** and **d**), non-transfected MUC1-positove and -negative cells (**c**) and control transfected Neo (**e**) cells were determined at 0 and 24 h. C1GT suppression increases caspase 3/7 activity in the MUC1-positive cells but has little influence in MUC1-negative cells. Data are expressed as mean±S.D. of triplicate determination of three experiments, ***P<0.001. In **c**–**e**, earlier apoptotic (Annexin-V positive and PI negative) cells show at the bottom right and late apoptotic (Annexin-V positive and PI-positive) cells show at the top right in each of the correlation plots. The numbers in each panel of the correlation plots show the percentage of the cells in total cell population.

We then assessed the anoikis response of these cells to suspended culture. It was found that in response to 24 h suspended culture, caspase 3/7 activity in the MUC1-negative neo cells was three times higher than in the paired MUC1-positive F3 cells (Figures 3a and b), confirming the role of MUC1 expression on cell resistance to anoikis as reported previously.^16^ To the MUC1-positive cells, shRNA suppression of C1GT (E7 and B7) resulted in higher caspase 3/7 activation in response to suspended culture than the control shRNA-treated (F2, C2) or non-transfected (F3) cells (Figure 3a). In the MUC1-negative cells, on the other hand, shRNA suppression of C1GT (sh-C1GT 3–4 produced no significant effect of cell caspase 3/7 activation in response to anoikis culture in comparison to either the shRNA control (sh-con 1–2) or parent MUC1-negative cells (Neo; Figure 3b). These results indicate that suppression of MUC1 Core 1 glycosylation markedly reduces MUC1-mediated resistance to anoikis.

When Annexin-V cell surface binding was assessed in response to 24 h suspended culture, the MUC1-positive F3 cells showed much less Annexin-V binding than the MUC1-negative (Neo) cells (32 *versus* 53% late apoptotic cells, respectively; Figure 3c). After shRNA C1GT suppression, the MUC1-positive cells (B7) showed 28% increase of Annexin-V binding in comparison to the control transfected shRNA cells (C8) (41 *versus* 32% late apoptotic cells, respectively; Figure 3d). On the other hand, suppression of C1GT (sh-C1GT-1) in the MUC1-negative (Neo) cells only slightly increased (5%) Annexin-V binding than the control transfected cells (sh-con-1; 61 *versus* 58%, respectively; Figure 3e) and the binding was also broadly similar as the control shRNA-treated cells. These results confirm the inhibitory role of MUC1 in cell resistance to anoikis shown previously^16^ and also support an active role of MUC1 *O*-linked sugar chains in MUC1-mediated cell resistance to anoikis.

### Suppression of C1GT increases ligand accessibility/binding to cell surface anoikis-initiating molecules

**Figure 4:**
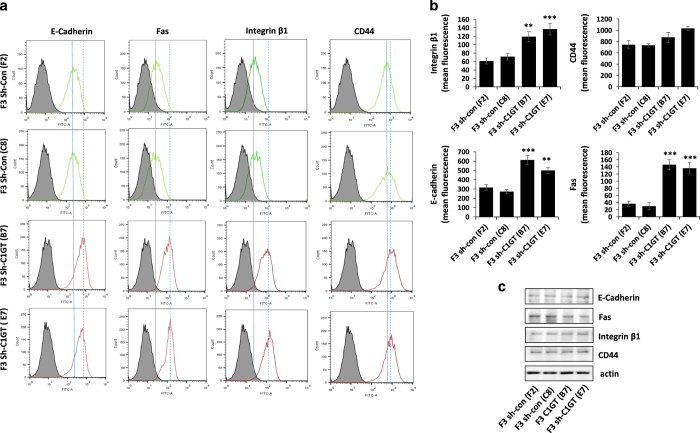
Effect of shRNA C1GT suppression on antibody accessibility to cell surface anoikis-initiating molecules. The shRNA C1GT transfected or shRNA control cells from the MUC1- positive F3 cells were incubated with antibodies against E-cadherin, integrin*β*1, CD44 or Fas. After washing and application of FITC-conjugated secondary antibody, the cells were analysed by flow cytometry (**a**, solid and dotted lines show the peak positions of control and shRNA C1GT transfected cells, respectively. Dotted line shifted to the left or right of the solid line reveals reduction or increase of molecule expression). Mean florescence intensity of the antibody binding are shown in **b**. Cells were also lysed and analysed by immunoblotting (**c**) for the expression of these molecules with the same antibodies as in **a**. **P<0.01, ***P<0.001.

Our previous study showed that MUC1-mediated resistance to anoikis is associated with MUC1 prevention of activation of the cell surface anoikis-initiating molecules, possibly by physically preventing the interaction of cell surface anoikis-initiating molecules with their ligands due to the great size of MUC1.^16^ It was found here that in comparison to the control shRNA cells (F2 and C8), antibody binding/accessibility to cell surface E-cadherin, Fas, integrin*β*-1, but not CD44, were all significantly increased in the shRNA C1GT suppressed cells (E7 and B7; Figures 4a and b). SDS-electrophoresis of cell lysates under denaturing conditions followed by immunoblotting with the same antibodies showed no difference in the expression of each of these molecules by these cells (Figure 4c). This indicates that the difference of antibody binding to these cell surface molecules shown in Figure 4a was not due to different expression of these molecules but likely due to differences in their accessibility. Suppression of C1GT in the MUC1-positive cells, which shortens the MUC1 *O*-linked carbohydrate structures, exposes the cell surface molecules and increases ligand-molecule interactions. This is in keeping with the role of MUC1 extracellular domain on inhibition of interaction of cell surface anoikis-initiating molecules with their ligands in anoikis shown earlier.^16^

### Suppression of C1GT inhibits anoikis induced by exogenous Fas-L

**Figure 5:**
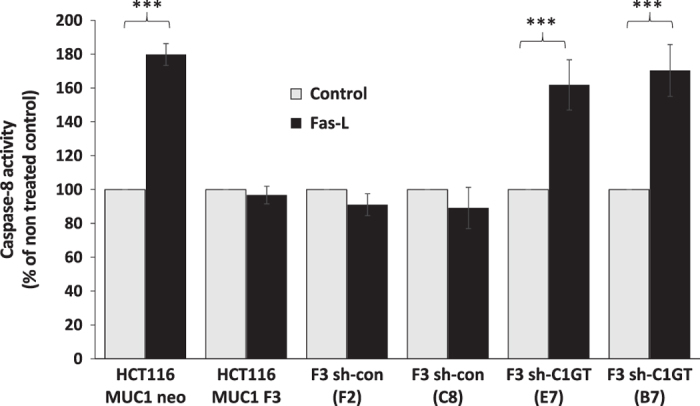
Effect of MUC1 expression and shRNA C1GT suppression on Fas-L-induced caspase-8 activation. The MUC1-positive (F3) and -negative (neo) cells and the shRNA control and shRNA C1GT transfected cells from MUC1-positive and -negative cells were cultured in the presence or absence of 100 ng/ml recombinant Fas-L in suspension for 2 h before the cell caspase-8 activity was determined by Caspase-8-Glo kit. Data are expressed as percentage to untreated cells (mean±S.E.M.) of triplicate determination of three experiments, ***P<0.001.

To test the role of MUC1 *O*-glycosylation on cell surface ligand-molecule interaction in anoikis induction, we compared the effect of exogenous addition of Fas-L on caspase-8 activation in cell response to anoikis culture of shRNA C1GT suppressed MUC1-positve and –negative cells. Interaction of Fas-L with Fas on the cell surface is known to induce anoikis/apoptosis through activation of caspase-8.^4,25^ It was found that introduction of exogenous Fas-L significantly increased the caspase-8 activity of the MUC1-negative (Neo) cells but had little influence on the MUC1-positive (F3) cells, again supporting the role of MUC1 expression on inhibition of Fas-L-Fas interaction-induced anoikis as previously reported.^16^ Suppression of C1GT in F3 cells (E7 and B7) resulted in significant increase of caspase-8 activity in cell response to anoikis culture, such an effect was however not observed in the control shRNA transfected cells (F2 and C8; Figure 5). These results again support a role of MUC1 *O*-glycosylation in MUC1-mediated anoikis inhibition by participating in preventing anoikis-initiating molecule interactions with their ligands on cell surface.

### Suppression of C1GT inhibits anoikis in MUC1-positive SW620 cells

**Figure 6:**
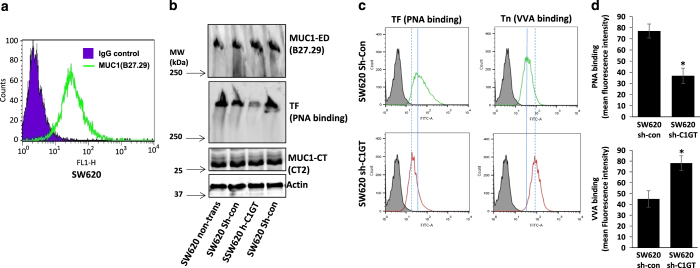
shRNA suppression of C1GT in human colon cancer SW620 cells. SW620 cells express very high level of MUC1 as determined by B27.29 anti-MUC1 antibody followed by analysis with flow cytometry (**a**). SW620 cells were transfected with shRNA C1GT or control shRNA and several stably transfected colonies were analysed by immune/PNA blotting with B27.29 antibody against the MUC1 extracellular domain (MUC1-ED) and CT-2 antibody against the MUC1 cytoplasmic tail/domain (MUC1-CT) and biotin-PNA (**b**). The cells were also analysed for cell surface TF and Tn expressions by biotin-PNA and biotin-VVA followed by flow cytometry (**c**). The mean florescence intensity of PNA and VVA binding in **c** were shown in **d**. Data are expressed as mean±S.D. of florescent intensity from duplicate determinations of 2 experiments, *P<0.05.

To further confirm the role of MUC1 *O*-glycosylation in MUC1-mediated cell resistance to anoikis, we stably transfected human colon cancer SW620 cells, which express high level of MUC1 (Figure 6a), with shRNA C1GT and control shRNA and generated C1GT stably suppressed cells. A 52% reduction of TF expression (PNA binding) was seen in the C1GT transfected cells (sh-C1GT) in comparison to the control transfected cells (sh-con) when analysed by PNA blotting (Figure 6b). A simultaneous increase of Tn (GalNAc-*α*) expression was also seen on the cell surface in the shRNA C1GT transfectants when analysed by flow cytometry (Figures 6c and d).

**Figure 7:**
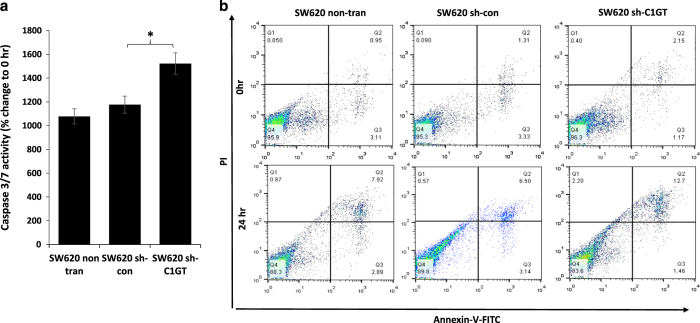
Effects of shRNA C1GT suppression on anoikis in SW620 cells. The shRNA control and shRNA C1GT transfected or non-transfected SW620 cells were cultured in suspension and the cellular caspase 3/7 activity was determined after 24 h (**a**). Annexin-V cell surface binding were determined at 0 and 24 h (**b**). Caspase 3/7 activity is presented as mean±S.E.M. of triplicate determination of three experiments. *P<0.05.

Suppression of C1GT expression (sh-C1GT) in MUC1-highly expressed SW620 cells resulted in significant increase of cell caspase 3/7 activity in response to 24 suspended culture in comparison to the non-transfected or control transfected (sh-con) cells (Figure 7a). The population of Annexin-V/PI-positive cells was also substantially increased in the sh-C1GT cells in comparison to the non-transfected or control transfected cells (12.7%, 6.5% and 7.9%, respectively, late apoptotic and necrotic cells; Figure 7b). These results also support the role of MUC1 *O*-glycosylation in cell resistance to anoikis.

### Suppression of C1GT in SW620 cells increases ligand accessibility to cell surface anoikis-initiating molecules and increases caspase-8 activity by exogenous Fas-L

**Figure 8:**
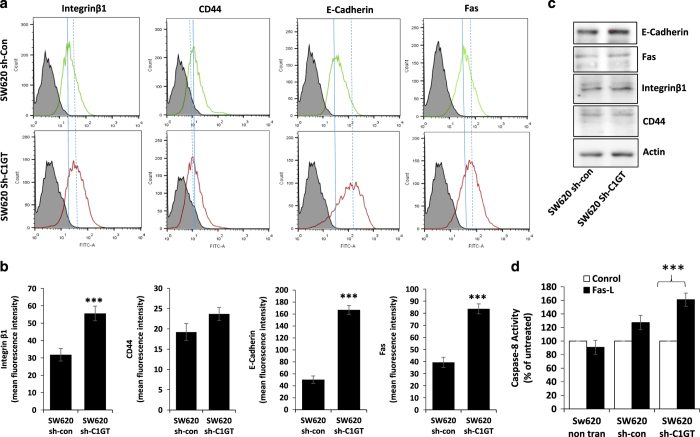
Effect of shRNA C1GT suppression increases antibody accessibility to cell surface anoikis-initiating molecules and enhances Fas-L-induced caspase-8 activity in SW620 cells. shRNA C1GT transfected or shRNA control transfected SW620 cells were incubated with antibodies against E-cadherin, integrin*β*1, CD44 or Fas. After application of FITC-conjugated secondary antibody, the cells were analysed by flow cytometry (**a**, solid and dotted lines show the peak positions of control and shRNA C1GT transfected cells, respectively. Dotted line shifted to the left or right of the solid line reveals reduction or increase of molecule expression). Mean (±S.D.) florescence intensity of the antibody bindings are shown in **b**. Cells were also lysed and analysed for the expression of these molecules by immunoblotting with the same antibodies (**c**). In **d**, the shRNA control and shRNA C1GT transfected SW620 cells were cultured in the presence or absence of 100 ng/ml recombinant Fas-L in suspension and the cell caspase-8 activity was determined after 2 h by Caspase-8-Glo kit. Data are expressed as mean±S.E.M. of triplicate determination of three experiments, ***P<0.001.

Ligand binding/accessibility to cell surface anoikis-initiating molecules was also compared in the shRNA control and shRNA C1GT suppressed SW620 cells. Antibody binding to cell surface E-cadherin, integrin*β*1 and Fas, like that in HCT116-F3 cells, was all increased in the C1GT suppressed cells in comparison to the control transfectants (Figures 8a and b). Again the expression levels of these cell surface molecules were the same among the transfectants (Figure 8c). When the caspase-8 activity was assessed in response to suspended culture, addition of exogenous Fas-L increased caspase-8 activity in the C1GT-suppressed but not the control cells (Figure 8d). Together, these results support the participation of MUC1 *O*-glycosylation in MUC1-mediated cell resistance to anoikis by preventing activation of cell surface anoikis-initiating molecules.

## Discussion

Here we have shown that partial inhibition of *O*-glycosylation by stable suppression of C1GT expression significantly increased anoikis of MUC1-positive, but not MUC1-negative, cells in response to suspended culture. Reduction of the MUC1 *O*-glycosylation increased ligand/antibody accessibility to cell surface anoikis-initiating molecules such as E-cadherin, integrin*β*1 and Fas and increased caspase-8 activity in response to exogenous introduction of Fas-L to suspended cells.

C1GT is a key glycosyltransferase in the biosynthesis of *O*-linked mucin type glycans, responsible for the formation of the Core 1-related complex *O*-glycans. Suppression of C1GT blocks Core-1 *O*-glycosylation and results in increased expression of the short glycans GalNAc*α* (Tn) and sialyl-Tn.^26^ Stable shRNA C1GT suppression to reduce MUC1 *O*-glycosylation is supported here by (1) substantial reduction of the MUC1 extracellular domain molecular weight size; (2) significant reduction of the TF disaccharide and (3) significant increase of the monosaccharide glycan Tn (Figure 1). As suppression of C1GT expression will also affect *O*-glycosylation on cellular glycoproteins other than MUC1, we also stably transfected the paired-MUC1-negative cells with shC1GT. Suppression of C1GT in the paired MUC1-negative cells reduced glycosylation of a number of cellular proteins (Figure 2). When the responses of these paired shRNA C1GT cells to suspended culture were compared, significant increase of anoikis in cell response to suspension culture occurred only in the MUC1-positive cells but not the MUC1-negative cells. This suggests that the increased anoikis observed in the MUC1-positive cells is attributed specifically to the reduced *O*-glycosylation of MUC1.

It is noted that elevated expression and activity of *N*-acetyl-glucosaminyltransferase-V (Mgat5), which catalyses the biosynthesis of *β*-1–6-linked GlcNAc in *N*-glycans and hence increases *N*-glycan branching,^27^ has been reported previously to promote anchorage-independent growth and inhibit anoikis in two hepatoma cell lines.^28^ Although *N*-glycans make only a small contribution to the overall glycosylation of mucin proteins like MUC1, their influence in the hepatoma cells is broadly in agreement with a role of glycosylation in anoikis shown in this study.

One of the very early events in anoikis initiation occurs on the cell surface through activation of the cell surface anoikis-initiating molecules through either conformation change, oligomerization or ligation with ligands.^3–5^ Ligand/antibody accessibility to the cell surface anoikis-initiating molecules such as integrin, E-cadherin and Fas is shown in this study to be substantially increased in the MUC1-positive cells after suppression of the MUC1 *O*-glycosylation through C1GT suppression. Caspase-8 activation in suspension culture in response to exogenous introduction of Fas-L is also significantly increased in the MUC1-positive but not MUC1-negative cells after C1GT suppression.

Thus, the extensive *O*-glycosylation of the MUC1 extracellular domain contributes to resistance to anoikis by preventing activation of cell surface anoikis-initiating molecules. This provides further insight into the molecular mechanisms of anoikis regulation and highlights the importance of cellular glycosylation in cancer progression and metastasis.

## Materials and methods

### Materials

The Caspase 3/7 Glo kits and Caspase-8 Glo kits were obtained from Promega (Southampton, UK). Recombinant Fas-L was from PeproTech (London, UK). Antibodies against CD44 (BBA10), integrin*β*1 (MAB17782), E-cadherin (MAB1838), Fas (AF2267) and Fas-L (AF126) were from R&D Systems (Abingdon, UK). FITC-Annexin-V/PI apoptosis detection kit was from Cambridge Biosciences (Cambridge, UK). Biotinylated peanut agglutinin (PNA) and biotinylated Vicia Villosa Lectin (VVA) were purchased from Vector Laboratories, (Peterborough, UK). FITC-conjugated anti-mouse antibody (115-095-146) was purchased from Jackson Immunoresearch Labs, West Grove, PA, USA). Chemiluminescence detection kits were from Thermo Scientific, (Rockford, IL, USA). Metafectene was from Biontex Laboratories (München, Germany). B27.29 anti-MUC1 antibody was kindly provided by Dr Mark Reddish (Biomira, Edmonton, Canada) and CT2 anti-MUC1 antibody was kindly provided by Prof Sandra Gendler (Mayo Clinic, AR, USA). ShRNA plasmid DNA for Core 1Gal-transferase (SHCLND-NM_020156-C1GALT, TRCN0000289384), control shRNA (SHC002v) and Non-enzymatic cell dissociation solution (NECDS) were from Sigma Aldrich (Dorset, UK)

### Cells

The MUC1-negative human colon cancer HCT116 and MUC1-positive SW620 cells were obtained from European Collection of Cell Culture (Salisbury, UK) and were cultured in McCoy’s5A medium. The cell lines were last authenticated by DNA profiling (DNA Diagnostics Centre, London, UK) in 2014. MUC1-expressiong HCT116MUC1-F3 and MUC1-negative HCT116MUC1-neo cells were obtained by stable transfection of HCT116 cells with MUC1-expressing or control vectors as described previously.^16^

### shRNA C1GT transfection

HCT116 cells were seeded in McCoy’s 5A media for 24 h until 60–70% confluent. ShRNA for C1GT1 or control shRNA (100 ng) was pre-mixed in 1 : 4 ratio with Metafectin transfection reagent in serum-free and antibiotic-free McCoy’s 5A media for 30 min before addition to the cells in antibiotic-free and serum-containing medium in 96-well plate. After 6 h culture at 37 °C, the culture media was replaced with selection media containing 10% serum and 0.5* μ*g/ml puromycin for 72 h. The surviving cells were released by trypsin, suspended in very low cell density and seeded into 96-well plates. Wells containing one cell were identified under microscope and allowed to proliferated before they were selected and analysed for TF, Tn or MUC1 expression by lectin/immune blotting and/or flow cytometry

### Immunoblotting

Cell lysates were separated by SDS-PAGE and transferred to nitrocellulose membrane. The membrane was incubated with biotinylated TF-binding PNA (2 *μ*g/ml), Tn-binding VVA (2 *μ*g/ml) or antibodies against MUC1 extracellular domain B27.29 (1 *μ*g/ml), MUC1 intracellular domain CT-2 (2 *μ*g/ml), E-cadherin (2 *μ*g/ml), CD44 (2 *μ*g/ml), integrin*β*1 (2 *μ*g/ml) or Fas (2 *μ*g/ml) for 16 h at 4 °C. These blots were washed 3 times with 0.05% Tween-20 in PBS before incubated with perioxidase-avidin (1 : 5000) and peroxidase-conjugated secondary antibody (1 : 3000) for 1 h. After six washes with 0.05% Tween-20 in PBS, the blots were developed using chemiluminescence Super Signal kit and visualised with Molecular Imager Gel Doc XR system (Biorad). The blots were striped by stripping buffer (62.5 mM Tris-HCl pH 6.7, 100 mM *β*-mercaptoethanol, 2% SDS) and re-probed by anti-actin antibody (1 : 3000) for protein loading.

### Assessments of cell anoikis

The assessments were conducted in cell suspension culture in poly-2-hydroxyethyl methacrylate (poly-HEMA)-coated plates as described previously.^16^ Briefly, 96- or 6-well plates were coated three times with 10 mg/ml poly-HEMA in 95% ethanol overnight. Cells were released by NECDS from the culture flasks, washed with PBS, resuspended to 5×10^5^ cells/ml in serum-free DMEM containing 0.5 mg/ml BSA and applied to the poly-HEMA-coated plates for various times at 37 °C. The cells were collected and the apoptotic (anoikis) cells were then measured either by FITC-Annexin-V/PI apoptosis detection kit with flow cytometry, or by the Caspase-Glo3/7 Assay kit according to the manufacturer’s instructions

### Flow cytometry to assess antibody binding to cell surface molecules

Cells were released from the culture plates by NECDS and fixed immediately with 2% paraformaldehyde for 15 min at room temperature. After washing with PBS, the cells were incubated with 5% goat serum in PBS for 30 min. The cells were re-suspended at 5×10^5^ cells/ml in 1% goat serum in PBS and incubated with biotin-PNA, biotin-VVA (2 *μ*g/ml) or antibodies against MUC1 extracellular domain B27.29 (1 *μ*g/ml), MUC1 intracellular domain CT-2 (0.5 *μ*g/ml), E-cadherin (0.5 *μ*g/ml), CD44 (0.5 *μ*g/ml), integrin*β*1 (0.5 *μ*g/ml), Fas (0.5 *μ*g/ml) or control mouse IgG on a rotation platform for 1 h at room temperature. After three washes with PBS, FITC- Avidin (1 : 500) or FITC-conjugated secondary antibody (1 : 500 in 1% goat serum in PBS) was applied for 1 h. The cells were washed three times with PBS before analysis by flow cytometry (BD FACS Canto II).

### Effect of exogenous Fas-L on caspase-8 activation

The cells were released by NECDS and seeded at 2×10^5^cells/ml in serum-free DMEM to poly-HEMA-coated 96-well plates with or without introduction of 100 ng/ml Fas-L for 0 and 2 h before the cellular caspase-8 activity was measured by Caspase-8-Glo Assay kit according to the manufacturer’s instruction.

### Statistical analysis

Unpaired *t*-test for single comparison, one-way analysis of variance (ANOVA) followed by Bonferroni correction for multiple comparisons (Sigmaplot v12 for Windows, Systat Software Inc, London, UK) were used where appropriate. Differences were considered significant when *P*<0.05.
